# Supplementary material for: TF2TG: an online resource mining the potential gene targets of transcription factors in Drosophila
Source: Genetics. 2025 May 2;232(1):iyaf082. doi: 10.1093/genetics/iyaf082 (PMC12774851; doi:10.1093/genetics/iyaf082)

Supplemental Figure 1. TF2TG use case. a) Binding sites from ChIP-seq data of sd and the transcription factors from the IMD signaling pathway (Rel, dl and Dif) in the transcriptional regulatory region of the cachectic ligand upd3, visualized on the TF2TG genome browser. b) CRMs of upd3 annotated by REDfly visualized on the FlyBase genome browser.

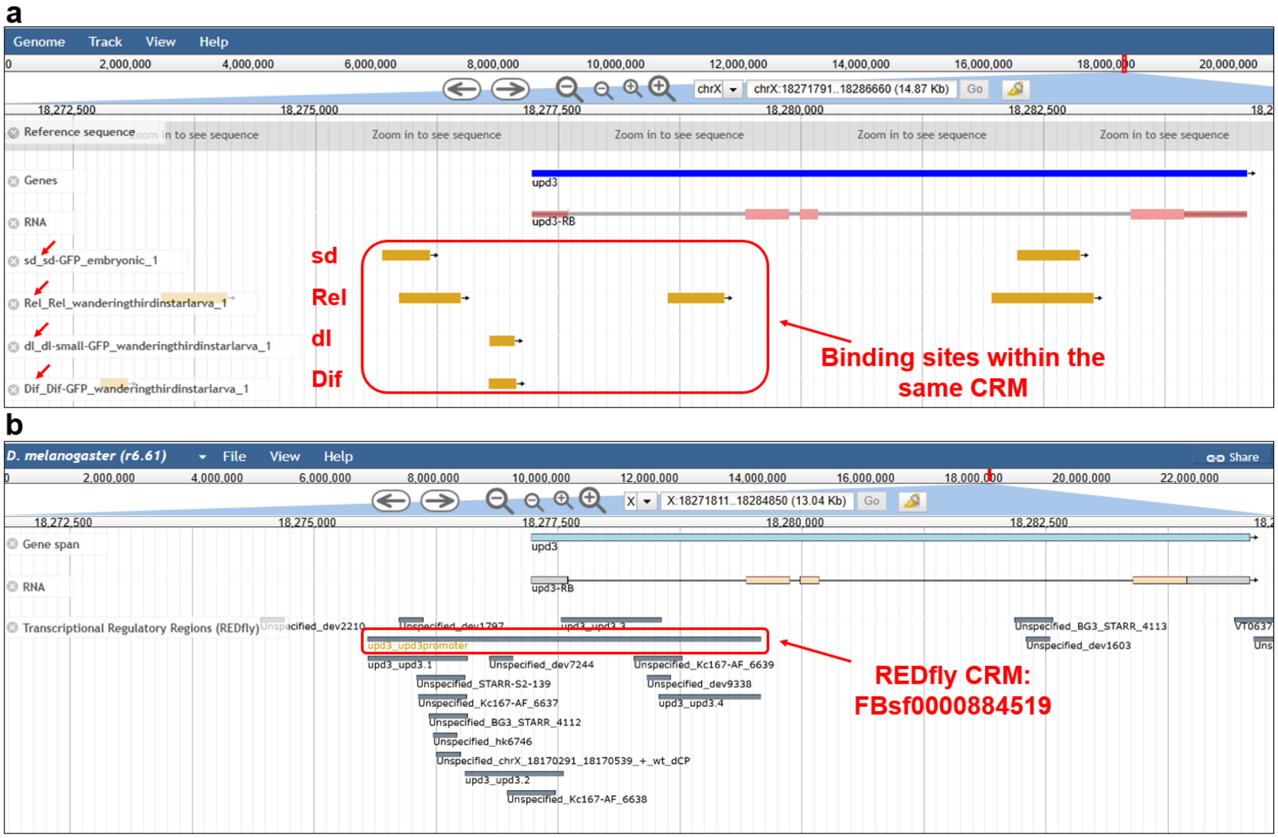

Supplement: iyaf082_Supplementary_Data [file iyaf082_supplementary_data.zip › Supplemental_Figure_1_GENETICS-2025-308063.pdf]
